# Is color data from citizen science photographs reliable for biodiversity research?

**DOI:** 10.1002/ece3.7307

**Published:** 2021-03-30

**Authors:** Alexandra Laitly, Corey T. Callaghan, Kaspar Delhey, William K. Cornwell

**Affiliations:** ^1^ Evolution and Ecology Research Centre School of Biological, Earth and Environmental Sciences University of New South Wales Sydney NSW Australia; ^2^ Max Planck Institute for Ornithology Seewiesen Germany; ^3^ School of Biological Sciences Monash University Clayton Vic. Australia

**Keywords:** birds, citizen science, color, photography, plants, spectrometry

## Abstract

1. Color research continuously demands better methods and larger sample sizes. Citizen science (CS) projects are producing an ever‐growing geo‐ and time‐referenced set of photographs of organisms. These datasets have the potential to make a huge contribution to color research, but the reliability of these data need to be tested before widespread implementation.
2. We compared the difference between color extracted from CS photographs with that of color extracted from controlled lighting conditions (i.e., the current gold standard in spectrometry) for both birds and plants. First, we tested the ability of CS photographs to quantify interspecific variability by assessing > 9,000 CS photographs of 537 Australian bird species with controlled museum spectrometry data. Second, we tested the ability of CS photographs to quantify intraspecific variability by measuring petal color data for two plant species using seven methods/sources with varying levels of control.
3. For interspecific questions, we found that by averaging out variability through a large sample size, CS photographs capture a large proportion of across species variation in plumage color within the visual part of the spectrum (*R^2^* = 0.68–0.71 for RGB space and 0.72–0.77 for CIE‐LAB space). Between 12 and 14 photographs per species are necessary to achieve this averaging effect for interspecific studies. Unsurprisingly, the CS photographs taken with commercial cameras failed to capture information in the UV part of the spectrum. For intraspecific questions, decreasing levels of control increase the color variation but averaging larger sample sizes can partially mitigate this, aside from particular issues related to saturation and irregularities in light capture.
4. CS photographs offer a very large sample size across space and time which offers statistical power for many color research questions. This study shows that CS photographs contain data that lines up closely with controlled measurements within the visual spectrum if the sample size is large enough, highlighting the potential of CS photographs for both interspecific and intraspecific ecological or biological questions. With regard to analyzing color in CS photographs, we suggest, as a starting point, to measure multiple random points within the ROI of each photograph for both patterned and unpatterned patches and approach the recommended sample size of 12–14 photographs per species for interspecific studies. Overall, this study provides groundwork in analyzing the reliability of a novel method, which can propel the field of studying color forward.

## Graphical abstract

Citizen science photographic data are a massive and continuously growing resource, with a largely untapped potential for breaking logistical barriers in color research. Using over 9,000 photographs of 537 species of Australian birds and two plant species, we assessed color accuracy and precision inter‐ and intraspecifically in comparison with spectrometry, the current gold standard.

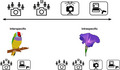

## 1 INTRODUCTION

Organism color is a visually remarkable, yet complex trait. Aspects of color are easily observed, and as such, much about its function, production, perception, and evolution is known. An organism's color can play vital roles in physiology, providing thermoregulatory (Caro, 2005; Stelbrink et al. 2019), photosynthetic, and photoprotective (Brenner & Hearing, 2008; Hirschberg, 2001) functions by regulating the absorption of light. More sophisticated functions of color evolved with the development of color vision in complex organisms, allowing coloration to be used in visual cues and signaling, such as the colorful plumage in birds for courtship and mating, or brightly colored flowers that attract animal pollinators (Dyer et al. 2012). Biological coloration arises from pigments or nanostructures, or an interaction between both mechanisms (Shawkey & D’Alba, 2017). Color is often used to study evolutionary processes such as selection and drift (Hoekstra, 2006). Increasingly, patterns at large scales, including color variation across biogeographic regions (Dalrymple et al. 2015) and throughout time (Zeuss et al. 2014), have proven to be interesting. In this line of color research, large sample sizes of observations through space and time are needed and these may prove difficult to obtain.

The study of color has traditionally been difficult for two main reasons: traditional observations of color have been subjective descriptions instead of quantitative measurements (Endler, 1990), and best practices of measuring color are expensive and time‐consuming (Leighton et al. 2016). Shifting from an abstract, qualitative description of color to quantitative data results in greater rigor in scientific studies, advancing our knowledge of color. Recent studies involving color in ecology and evolution have used spectrometry (Dalrymple et al. ,^2015^, ^2018^; Delhey, 2015; Shrestha et al. 2013), photography (Dalrymple et al. 2018; Shrestha et al. 2013; Tapia‐McClung et al. 2016), or in some cases scanning of illustrations (Dale et al. 2015; Pinkert et al. 2017; Stelbrink et al. 2019). The success of these approaches for different questions suggests that different aspects of color research will continue to draw from different data sources.

There are still logistical issues which create limitations to the use of these modern methods in ecology and evolution. While the use of spectrometers has become the standard in measuring color objectively (Badiane et al. 2017; Endler, 1990; Johnsen, 2016), it is still expensive (Byers, 2006) and technically difficult (Johnsen, 2016). Spectrometers can produce greatly varied measurements with different lighting conditions, and from variation in either angle of illumination or angle of observation (Johnsen, 2016), factors that are rarely kept constant across different setups. While commercial photography offers a more affordable, practical, and accessible alternative to spectrometry, there is often a great deal of information loss compared to spectrometry measurements. Nonetheless, the sampling process associated with both methods is still most likely limited to existing specimens or biodiversity at a particular time and place. Large‐scale macroecological research thus requires that scientists invest much time, labor, and funding to be able to gather sufficient data across spatial and temporal scales (Pocock et al. 2017).

One potential way to obtain biological data at broad spatial and temporal scales is by using citizen science (CS). CS, which refers to the contribution of scientific data from people outside of the professional scientific community regardless of citizenship status, mostly comprises data collection that requires little to no additional training or equipment (Rotman et al. 2012). This effort harnesses observers who already engage in hobbies and activities like birdwatching (Silvertown, 2009), bug‐catching (Yoshioka, 2013), and wildlife photography (Nowak et al. 2020), as well as seeks to spread both scientific contribution and engagement with nature. Compared to traditional data collection efforts, a major advantage of CS efforts is in the large number of contributors, thus expanding the temporal and geographic scope of data collection (Pocock et al. 2015) and allowing scientists to focus on analysis rather than collection of data (Cohn, 2008).

Today, the ubiquity of smartphones with cameras and GPS capabilities has paved the way for the growth of online CS projects composed of photographic data with embedded spatiotemporal metadata, collectively making up a massive database of individual phenotypic information. Two successful platforms which have gathered millions of photographed individuals are iNaturalist (www.inaturalist.org) with over 50 million observations and photographs across all living taxa, and the Macaulay Library (www.macaulaylibrary.org) with over 20 million photographs of birds. Given the recent surge in available data (i.e., photographs) the application of such CS photographs in the field of color research (Atsumi & Koizumi, 2017; Austen et al. 2018; Drury et al. 2019; Kerstes et al. 2019; Leighton et al. 2016; Moore et al. 2019; Parkinson et al. 2016) is still relatively new. The lack of control and standardization in CS photography subjects it to numerous potential issues. For example, color appearance and measurement in photographs are susceptible to variation from the effects of different shutter speeds, lighting conditions, noise (Jackowski et al. 1997), camera exposure levels, and specifications of the individual cameras used (Byers, 2006). Additionally, these effects vary depending on the subject of the photograph, likely associated with camera sensitivity to the optical properties of different colors and surfaces. Therefore, prior to the broad‐scale application of CS photographs in color research, there needs to be a fair assessment and accounting of its limitations, as well as a quantification of how color information from CS photographs corresponds with color information from controlled spectrometry.

**FIGURE 1:**
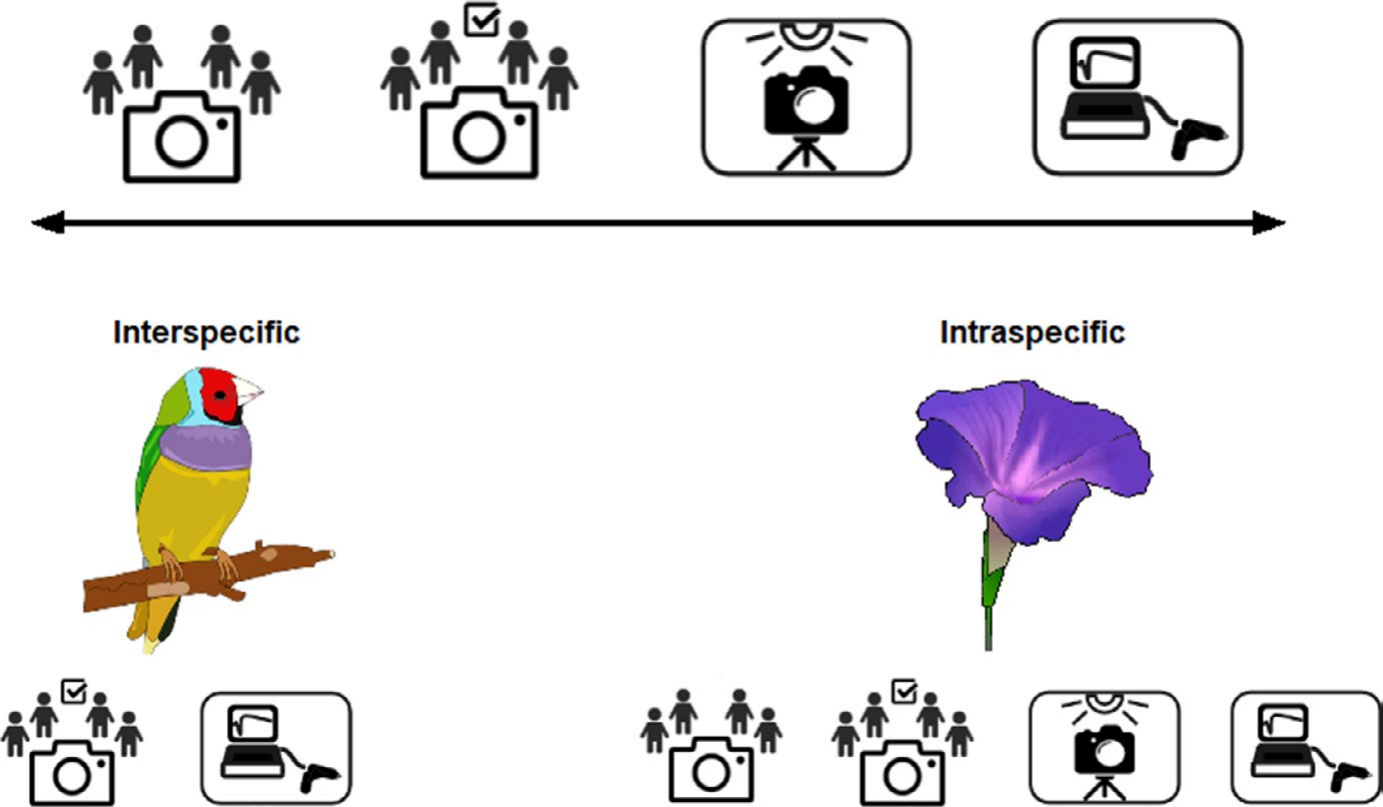
The spectrum of control in the study of color is represented by citizen science photographs, high‐quality citizen science photographs, controlled photography, and controlled spectrometry. Sample size generally decreases as level of control increases. We analyzed interspecific variability in birds, comparing color between high‐quality citizen science photographs and controlled spectrometry. We also analyzed intraspecific variability in plants, comparing color across all four levels of control

Our overall objective was to assess the ability of using CS photographs to quantify color for both interspecific (using birds as a study system) and intraspecific (using plants as a study system) questions. We first tested the ability of using CS photographs to make interspecific comparisons (i.e., among species) using color information for >500 bird species. We then tested whether specimen age, patterning in the plumage, or different subjective color families influence color measurement. Second, we quantified the ability to use CS photographs to capture intraspecific variability in color, by analyzing color across a spectrum of methodological control, ranging from spectrometry to CS photographs for two distinct and different plant species (Figure 1). We hypothesized that the variability in color measurements would increase from controlled (i.e., spectrometry) to uncontrolled (i.e., CS photographs) methods. For both objectives, our analyses were conducted in three common color spaces: RGB (red green blue), HSV (hue saturation value), and CIE‐LAB (abbreviated as Lab). Ultimately, our work will demonstrate the largely untapped potential of CS photographs and establish the reliability of this novel method for use in biodiversity research.

## 2 MATERIALS AND METHODS

### 2.1 Interspecific: Birds

#### 2.1.1 Species selection and sampling of citizen science photographs

We used photographs from the Macaulay Library CS project. The Macaulay Library (https://www.macaulaylibrary.org/) houses over 20 million photographs of birds, contributed by volunteer birdwatchers all over the world. Each photograph is rated by the birdwatching community based on their quality (see: https://help.ebird.org/customer/en/portal/articles/2665949‐photo‐quality‐rating‐guidelines?b_id=1928), ranging from 1 (barely identifiable to species) to 5 (excellent, in‐focus photo). For Australian species with a large number of photographs (>50), we requested a random subsample of 4‐ and 5‐star photographs (maximum of 50 per species) from the Macaulay Library. For any other species, we requested all photographs greater than a 3‐star rating. We obtained 22,754 photographs of 563 species which we then manually filtered again to exclude poor photographs that we considered to have been inaccurately rated or that were clearly duplicates.

For each species, all photographs were sorted by visual appearance into adult or juvenile, male or female if sexually dichromatic, and breeding or nonbreeding plumage where applicable, using Pizzey and Knight (2012). To limit the influence of intraspecific color variation on our analysis, we excluded the following photographs: (a) juveniles; (b) nonbreeding plumage birds; (c) females with obviously different coloration; and (d) birds in molt or in between forms. All morphs or races for a species were included as long as they were either all sexually dichromatic or all sexually monochromatic.

We focused on the upper breast because it is commonly used in comparisons of bird colors (Dale et al. 2015; Mcqueen et al. 2019) and because it is commonly visible in photographs. The photographs were filtered further to obtain only those with a visible upper breast patch, large enough that measuring its color would be feasible (detailed in Section 2.1.3). Patterned patches were treated no differently from solid patches, and with color‐blocked patches, one solid color was chosen to be the region of interest (ROI; Figure S1). Information on each bird species and its upper breast plumage was recorded to note where multiple morphs are present and if the ROI is patterned or part of a color‐blocked patch.

#### 2.1.2 Measuring colors in citizen science photographs

For each CS photograph that met the criteria above, we used *colorZapper* (Valcu & Dale, 2014) to retrieve color information in both RGB and HSV color spaces. Three random points were selected within the ROI of each photograph (Figure S1). Multiple colors in patterned patches (see Figure S1) were not treated as separate colors because (a) in many photographs it is difficult to define the borders between spots/streaks and background color (unlike in color‐blocked patches where there is a more distinct separation between solid colors), and (b) we tried to measure patches as similarly as possible to how spectrometry is conducted, which measures an average color within a patterned area. A maximum of 25 photographs per species was measured, and only one bird was measured for every photograph for cases in which multiple individuals were present in a photograph. Because of the potential for misinterpreting the location of the upper breast on some species, we inspected the RGB values from the museum spectral measurements in comparison with our data to ensure we measured the same part of the bird.

#### 2.1.3 Processing bird spectral measurements

We used spectral data on 555 Australian bird species from Delhey (2015). These data consist of reflectance spectra of plumage on museum specimens taken using a spectrometer under controlled lighting conditions (for detailed methods, see Delhey, 2015). After resolving taxonomic differences, we were left with a total of 537 species that were in common with species obtained from the Macaulay Library. Because the spectral data also encompassed birds of both sexes and a total of 17 plumage patches, this was again filtered to only include measurements of the upper breast plumage of male birds. We converted the spectral measurements into RGB values using the R library *pavo* (Maia et al. 2013). We also used a psychophysical model of avian color vision (see detailed methods in Supplementary Methods).

#### 2.1.4 Statistical analyses

Our analyses were conducted in R version 3.6.2 and used the *tidyverse* workflow (Wickham et al. 2019). We performed analyses at two levels: a species mean (*N* = 537) and the individual photograph (*N* = 9,441). The three measurements within the ROI (Figure S1) were averaged to obtain a mean R, G, and B value for each photograph. Firstly, we performed an overall analysis on the relationship between CS and museum color measurements at the species level. We plotted species mean values for CS against corresponding species mean values for museum and ran linear models to obtain *R^2^* values for each color component in RGB space. The same analyses were then done at the individual (photograph) level, which used individual values for CS and corresponding species mean values for the museum measurements.

Because we had sampled varying numbers of photographs per species ranging from 1 to 25, we explored whether increasing sample size produced lower residuals. Species‐level CS measurements were regressed against museum species mean measurements to obtain these residuals. We visualized the effect of increasing sample size on the mean color estimate by taking the absolute values of the residuals and averaging these for each number of photographs per species (1–25). We also tested several hypotheses to identify other explanatory variables. As the museum data were obtained from specimens of varying ages, we analyzed whether specimen age affects color. This involved using linear models on residual plots to obtain *R^2^* and *p*‐values for R, G, and B. We also analyzed whether patterned patches/ROIs measure differently from unpatterned ones. We then considered if RGB values within different color families (e.g., white, pink, or yellow) are captured differently by commonly used cameras. First, species were categorized subjectively into color families by which color(s) appear on their upper breast ROI. Those with multiple colors on their ROIs (e.g., patterned patches, multiple forms) were included in multiple color families (Table S1). Linear models were then run on plots of individual CS measurements versus museum species mean measurements to obtain residuals. Standard deviations were calculated for each species. These were displayed in box plots to show differences in accuracy and precision across different color families.

We also analyzed the data in two other color spaces, HSV and Lab. The latter color space is unique in that it separates chromatic (the wavelength of photons, *a* and *b*) from achromatic (the amount of photons, *L*) variation. As we had done with the RGB measurements, we averaged the HSV measurements from *colorZapper* to obtain mean values for each photograph. Lab equivalents were obtained by converting RGB measurements using *patchPlot* (Bruneau, 2013), followed by averaging. Museum data had to be converted for both HSV and Lab, using functions built into base R version 3.6.2 (R Core Team, 2019) and *patchPlot* (Bruneau, 2013). HSV values were further analyzed for precision by color family for both CS and museum data. Finally, we ran models predicting species means from museum specimens in the achromatic part of the bird visual space using species means from CS measurements in the achromatic part of the Lab space, as well as each of the x, y, and z dimensions for each eye type (U and V) using three linear models with the Lab chromatic components of *a* and *b* as predictors.

### 2.2 Intraspecific: Plants

#### 2.2.1 Species selection and sampling

We used two common species in this study, representing different subjective colors: *Macroptilium atropurpureum* and *Senna pendula*. Photographs were manually filtered for quality and whether the photographed flower is large enough to measure its color without difficulty. An additional step was performed to separate high‐quality photographs from lower quality photographs, using eBird guidelines adapted for plants (see above).

#### 2.2.2 Spectrometry

Spectrometry was performed inside an enclosed setup (Figure S2) to minimize the influence of outside lighting sources. This consisted of a cardboard box with a halogen 46W downlight positioned centrally over a matte black (Johnsen, 2016) specimen platform and a stand which secured the ASD FieldSpec4 fiber optic cable at a 30‐degree angle of observation, which we found to minimize issues with gloss and shading. Only one petal was measured for each flower. The petal was laid down as flat as possible on the platform, at various optimal distances—depending on the size and shape of the petal—from the fiber optic cable tip, ensuring that (a) the petal fully encompassed the field of view and (b) there was no shading from the cable tip within the field of view. Three measurements of reflectance for wavelengths 350–2,500 nm were recorded for each petal without moving. An opening on the front which allowed for the movement and positioning of flower petals was fully covered when taking all spectral measurements. As with bird spectral measurements in Section 2.1.2, reflectance values were then converted into RGB using *pavo* (Maia et al. 2013) on R.

#### 2.2.3 Photography and processing flower measurements

Controlled photography was performed at four levels: (a) Olympus TG‐5 camera set to microscope with no flash, (b) Olympus TG‐5 camera set to microscope with fill‐in flash, (c) iPhone X camera on default settings with no flash, and (d) iPhone X camera on default settings with flash. Both of these cameras are among the most commonly used on the iNaturalist platform by citizen scientists. Only one photograph was taken for each treatment for every petal, which was positioned exactly as detailed in Section 2.2.2, although the opening was not covered when taking photographs to accommodate the handling of cameras. As with bird photographs in Section 2.1.3, we obtained color measurements of three random points for each photograph (CS and controlled) using *colorZapper* (Valcu & Dale, 2014) on R.

#### 2.2.4 Statistical analyses

To prepare our data for analysis, RGB values from 2.2.2 were averaged for each individual flower, and RGB values from 2.2.3. were averaged for each photograph. These were then plotted together to visualize and analyze differences in color measurements across all treatments. Standard deviations in RGB and Lab were computed for each treatment. We then carried out Bartlett's test to check for variances across treatment groups, followed by a Kruskal–Wallis test to determine whether the differences across treatment groups are statistically significant.

## 3 RESULTS

### 3.1 Interspecific variability

**FIGURE 2:**
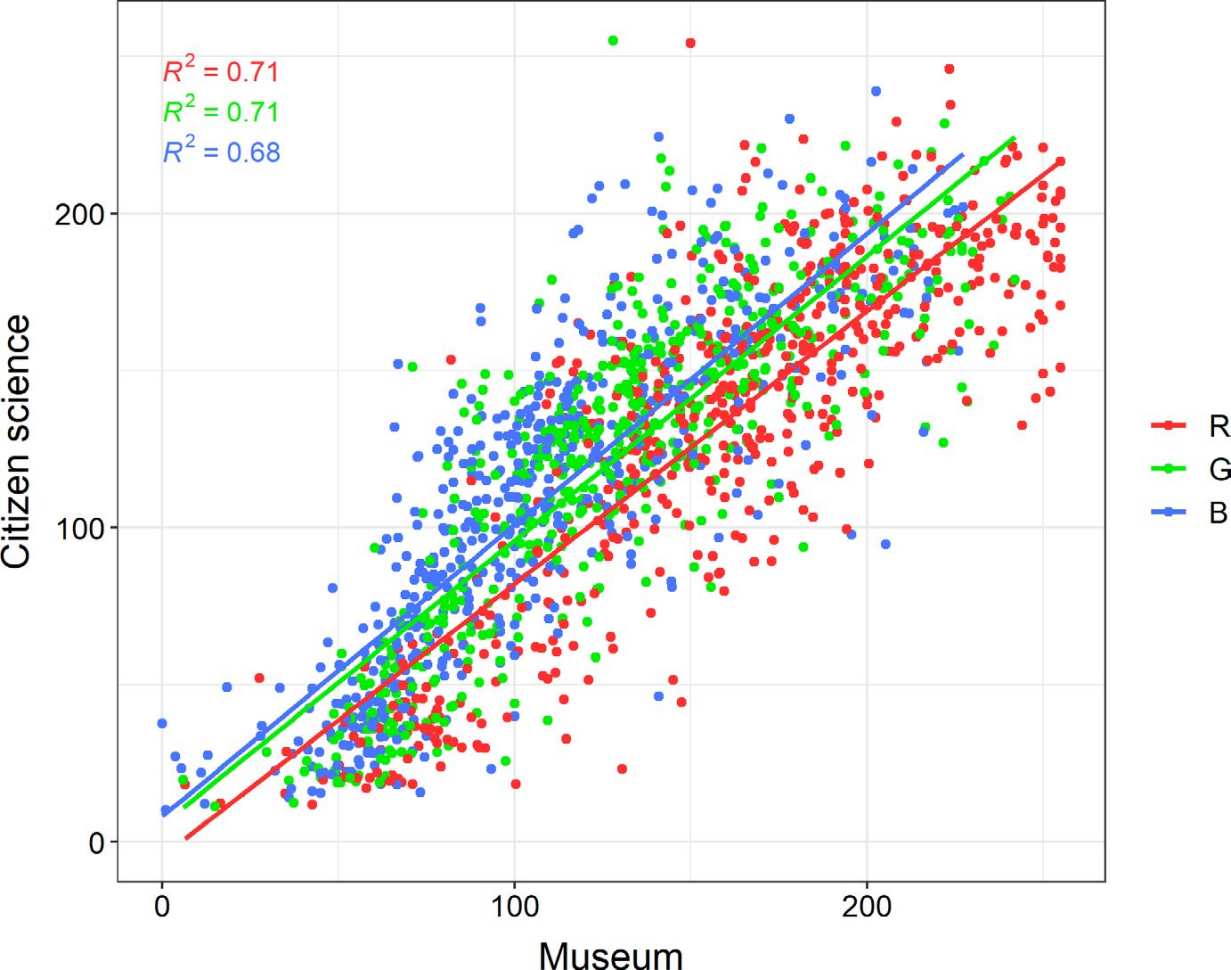
Linear model of the relationship between citizen science and museum colors in RGB space. Each point is a species mean in citizen science data plotted against the corresponding species mean in museum data

**FIGURE 3:**
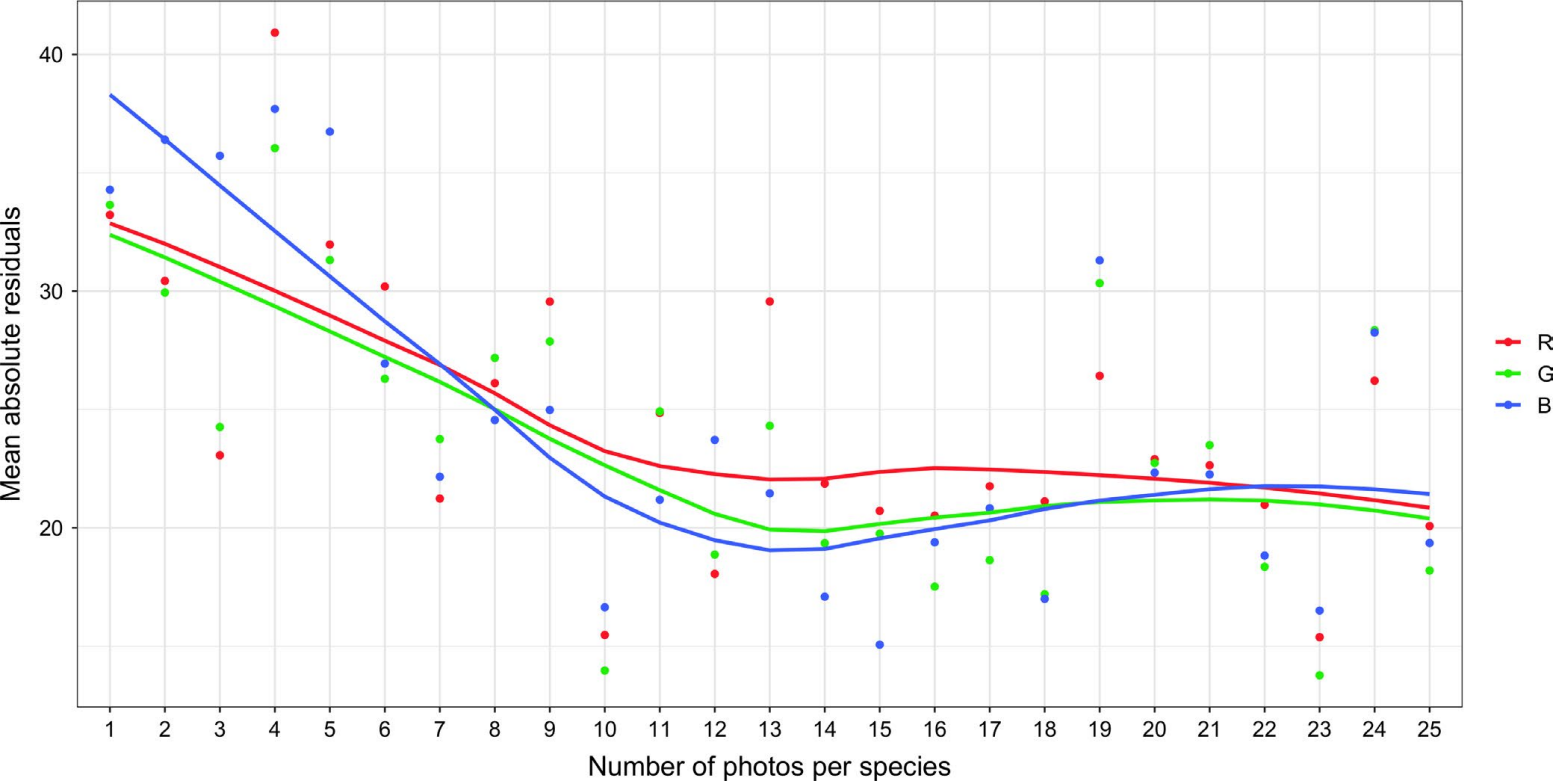
Relationship between sample size and precision. RGB measurements in the citizen science data were averaged at the species level and plotted against corresponding species mean values in the museum data to obtain residuals. Absolute residuals were then averaged for each number of photographs per species from 1 to 25

We analyzed a total of 9,441 CS photographs of 537 bird species. The mean number of photographs for a species was 17.6 ± 8.1. We found a strong relationship between CS and museum color measurements at the species level, with *R^2^* values of 0.71, 0.71, and 0.68 for R, G, and B, respectively (Figure 2). When the relationship was analyzed with individual CS photographs, we found lower *R^2^* values of 0.48, 0.47, and 0.46 for R, G, and B, respectively (Figure S3). Museum measurements on average measured noticeably higher for R, while CS measurements on average measured higher for B (Figure S4). Increasing the number of photographs measured for each species appeared to have a decreasing effect on residuals, however the increase in precision plateaued at approximately 12–14 photographs (Figure 3).

**FIGURE 4:**
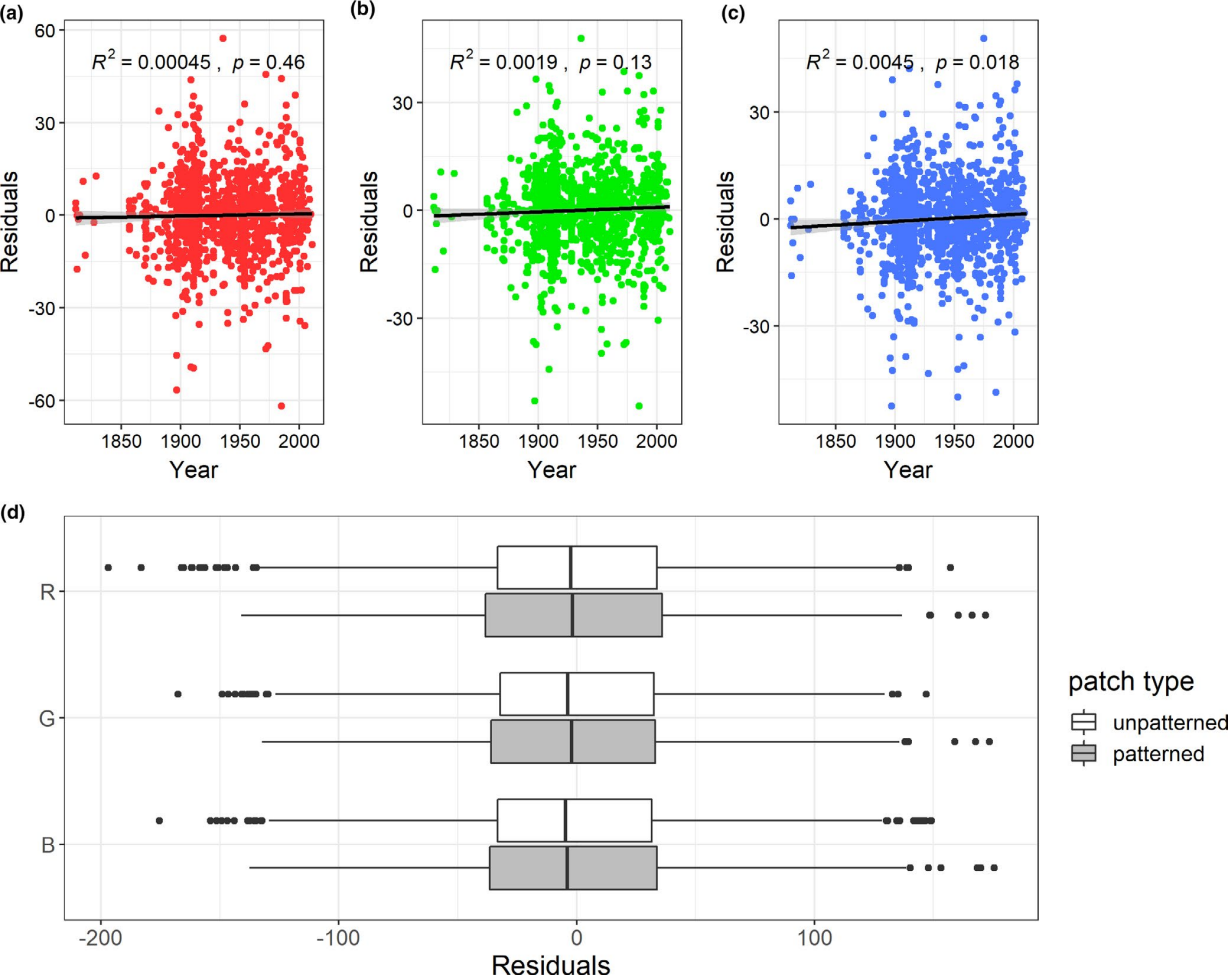
Panels (a), (b), and (c) show the relationship between color and specimen age. Residuals were obtained by plotting individual specimen measurements against species mean measurements. Panel (d) shows differences in bias in patterned and unpatterned patches; there was no significant difference for any of the three contrasts (*p* = 1). Residuals were obtained from plotting individual citizen science measurements against corresponding museum species mean measurements

**FIGURE 5:**
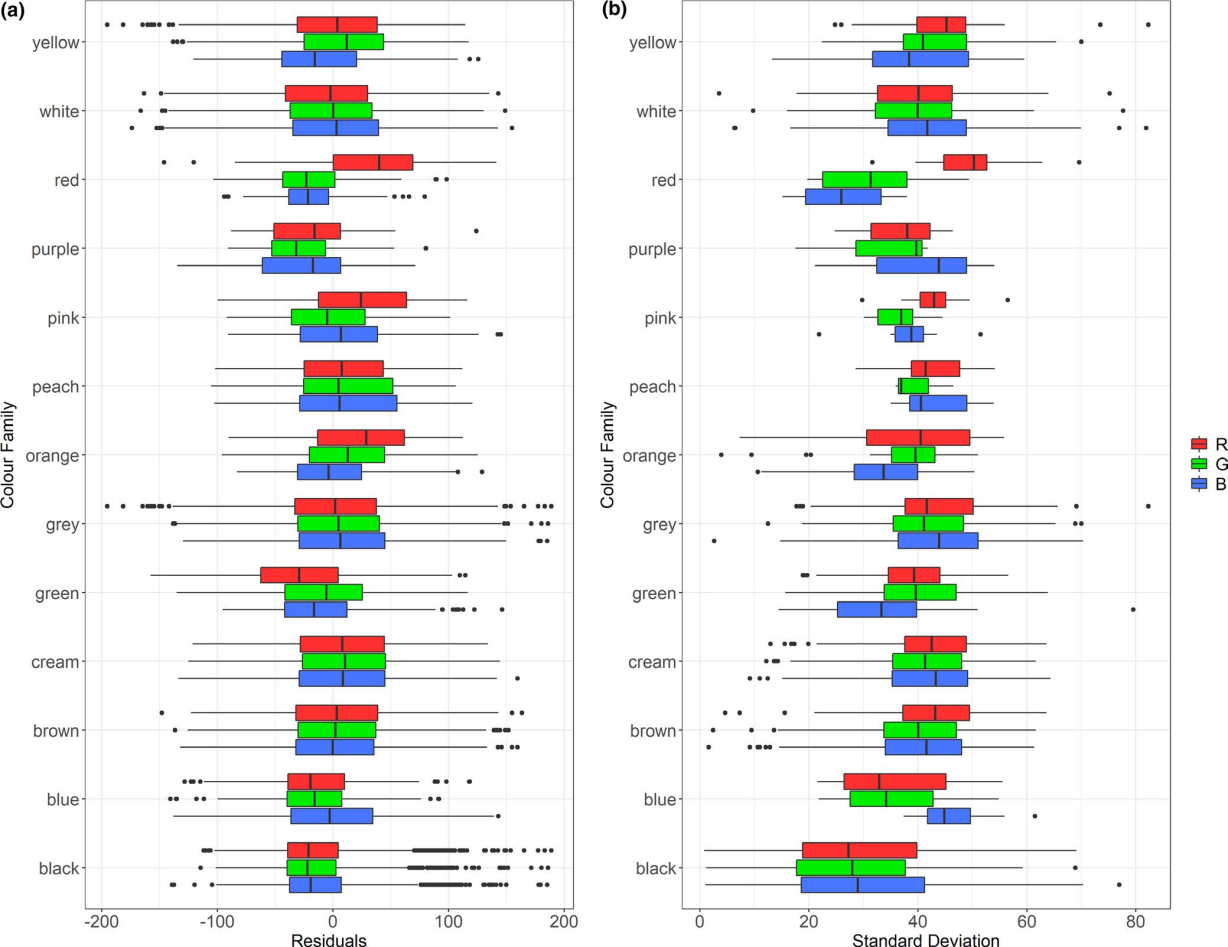
(a) Box plot showing differences in bias by color family. Residuals were obtained from plotting individual citizen science measurements against corresponding museum species mean measurements. (b) Box plot showing differences in variability by color family. Standard deviations were calculated at species level

Residuals for R and G in RGB space do not seem to be affected by specimen age, however, there is a weak effect found in B (Figure 4). There is no observable bias in patterned patches (Figure 4), suggesting that our method of measuring color in CS photographs is comparable to the spectrometry method applied in Delhey (2015). R measurements in red ROIs are higher on average in CS photographs, especially noticeable because of the considerably lower G and B measurements (Figure 5). R measurements for red patches also stand out for high imprecision, varying greatly across photographs (Figure 5). Additionally, black ROIs display a large range of standard deviations (Figure 5).

For all three color spaces, *R^2^* values improve considerably at the species level in comparison with the individual level (Table 1). RGB space in particular has *R^2^* values at the species level about 1.5 times of *R^2^* values at the individual level. Results in the RGB color space are very similar to results in the Lab color space: overall *R^2^* values are 0.72, 0.75, and 0.78 for L, a, and b, respectively. While all other *R^2^* values at the individual level remain below the 0.5 mark, the *a* and *b* dimensions in Lab color space have relatively higher correlation: 0.68 and 0.62, respectively (Table 1). We found mixed results in HSV, with strong *R^2^* values for S and V at 0.61 and 0.71, but 0.15 for H (Table 1; Figure S5). H values are very variable especially in CS data across almost all color families, and pink and purple ROIs measure poorly for H even under controlled spectrometry in museum data (Figure S6).

**TABLE 1:** *R^2^* values computed for citizen science versus museum measurements in three different color spaces (RGB, HSV, Lab) at species and individual levels

Color space	Component	*R* ^2^ values	
		Species level	Individual level
RGB	R	0.71	0.48
	G	0.71	0.47
	B	0.68	0.46
HSV	H	0.15	0.069
	S	0.61	0.45
	V	0.71	0.46
Lab	L	0.72	0.48
	a	0.75	0.68
	b	0.78	0.62

In our analysis of how well color in CS photographs corresponds with measurements in bird visual space, we found a strong relationship between the achromatic components from each space, with an *R^2^* value of 0.73 (Table 2). Chromatic components correspond well for both U‐ and V‐type eyes in y and z, all with *R^2^* values exceeding 0.7. However, the relationships are weaker for both eye types in x, with values of 0.1 and 0.46, respectively.

**TABLE 2:** *R^2^* values computed for citizen science measurements in Lab space versus museum measurements in bird visual space

*R* ^2^ values			Lab space	
			L	a + b
Bird visual space	DL		0.73	
	U‐type	x		0.1
		y		0.74
		z		0.76
	V‐type	x		0.46
		y		0.79
		z		0.78

Note

For Lab, a and b together represent the chromatic component. Relationships are either between two chromatic components or two achromatic components, one from each space.

### 3.2 Intraspecific variability

**FIGURE 6:**
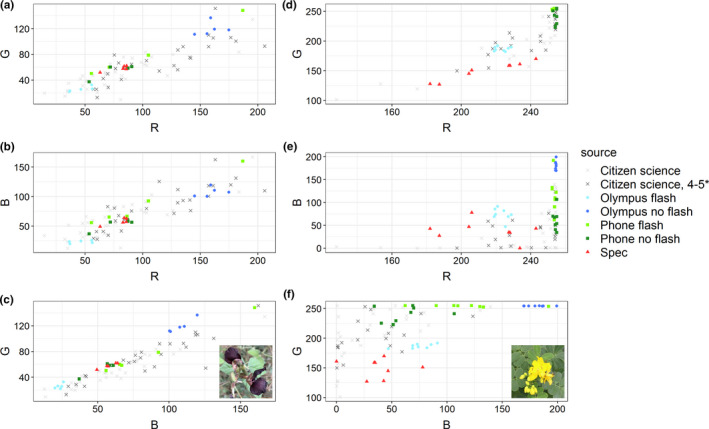
Plots for *Macroptilium atropurpureum* (a–c) and *Senna pendula* (d–f) displaying RGB measurements across 7 treatments. Flower images are CC0

We analyzed 48 CS photographs and 8 individual specimens for *Senna pendula*, and 62 CS photographs and 5 individual specimens for *Macroptilium atropurpureum*. RGB values for lower quality CS photographs show high variability (Figure 6). We measured extremely high R and G values in *Senna pendula*, especially for “Olympus no flash,” which appear condensed at 255 (Figure 6), indicating a saturation issue (Stevens et al. 2007); therefore, *Senna pendula* data were excluded from further analyses.

In RGB space, the variability of CS photographs is higher than all other methods for *Macroptilium atropurpureum* except for “Phone flash,” while “Olympus flash” showed the lowest variability—even less than spectrometry (Table 3). However, in Lab space, where achromatic variation is separated from chromatic variation, the high standard deviation in “Phone flash” was found to be mostly from the lightness (*L*) component (Table 4). Additionally, spectrometry measurements had the lowest variability in all three Lab space components, with values of 2.29, 2.87, and 1.23, respectively. Our subjective rating system which separated low from high‐quality photographs did not improve variability in the latter group. The variances across treatment groups for R, G, and B are different (*p* <.001 for all six tests). Following that, all Kruskal–Wallis tests had *p*‐values of less than .001.

**TABLE 3:** Standard deviations computed for Figure 6, showing values obtained for *Macroptilium atropurpureum* and *Senna pendula* across seven treatments

Species	Source	sd_red	sd_green	sd_blue
*Senna pendula*	Citizen science	33.16	45.65	42.88
	Citizen science, 4–5*	17.42	29.28	32.02
	Olympus flash	3.84	3.59	13.52
	Olympus no flash	0.19	0.18	10.11
	Phone flash	0.83	0.98	37.63
	Phone no flash	0.80	12.96	22.49
	Spec	22.34	15.66	21.72
*Macroptilium atropurpureum*	Citizen science	38.45	25.18	30.34
	Citizen science, 4–5*	43.78	31.21	34.84
	Olympus flash	9.74	4.14	2.33
	Olympus no flash	10.77	10.30	7.95
	Phone flash	51.41	40.02	42.43
	Phone no flash	15.46	10.03	9.63
	Spec	9.77	4.20	5.83

Note

Each component of RGB was measured separately.

**TABLE 4:** Standard deviations computed for *Macroptilium atropurpureum*, in Lab space

Species	Source	sd_L	sd_a	sd_b
*Macroptilium atropurpureum*	Citizen science	12.92	9.81	7.14
	Citizen science, 4–5*	14.46	11.21	8.58
	Olympus flash	4.57	4.63	4.51
	Olympus no flash	5.22	6.57	2.58
	Phone flash	19.04	6.43	2.41
	Phone no flash	8.49	5.18	2.86
	Spec	2.29	2.87	1.23

## 4 DISCUSSION

Using photographs of both birds and plants, we demonstrate the potential use of CS photographs in the future of color research for both interspecific and intraspecific ecological and biological questions. For our interspecific objective (i.e., taking a mean across many samples per bird species), we found strong relationships between CS and museum color measurements in both RGB and Lab color spaces, suggesting that CS photographs capture a significant amount of color information for interspecific studies. For our intraspecific objective (i.e., using photographs of two plant species), we found that along the spectrum of control, intraspecific variability was overall greatest for CS photographs and lowest for spectrometer measurements. We demonstrated that improvement in the precision of a species' mean color estimate slows greatly after 12–14 photographs, suggesting that a larger sample size to a point does help to average out method dependent variability across individual photographs, albeit to a lesser extent compared to interspecific studies. Moreover, given the growing popularity of CS—and contributions of photographs—this is likely a feasible sample size for many questions. In this study, the mean number of photographs per bird species was 17.6, but we recognize that available sample sizes will depend on the question.

Using photographs of organisms in nature is closer to the functional purpose of the color (i.e., attracting a mate or a pollinator) compared to museum measurements, but the natural setting introduces a great deal of variability in the measurement. Differences in lighting and equipment can contribute greatly to noise and imprecision, obscuring true results when studying color in an ecological context. In our analyses, we found strong relationships at both the individual and species levels, despite the variability, in RGB space and even more so in Lab space. Interestingly, because the Lab color space separates chromatic (*a* and *b*) from achromatic components (*L*), our analysis at the individual level shows that the variation is more related to the combination of how brightly lit a patch is and the capture of light by individual cameras versus chromatic differences (Table 1).

Aside from the level of control, we also evaluated three factors—patterning, specimen age, and color families—for their effect on the variability of color measurements. None of these three had strong effects, but there were a few exceptions such as a weak effect of the age of a specimen in B values, although this may be due to a low sample size (mean of 2.62 per species). Whether bird specimens accurately preserve color in living birds is an area of research with mixed results (Armenta et al. 2008; Doucet & Hill, 2009; McNett & Marchetti, 2005), but there was minimal effect of specimen age in this specific study. Future research should confirm these comparisons between CS photographs and museum specimens for a larger number of samples of museum specimens, given the potential of conflating factors such as the variability between individuals that exist in nature. Overall, we did not find substantial differences across the subjectively assigned color families, although there are noticeable points in red and black. All museum measurements on average are higher in *R* values, but red ROIs specifically measure higher for R in CS photographs, which are also the most imprecise values. This could be due to saturation in photographs and camera processing; however, within our sample we found that cases of R‐saturated red patches were rare. Black ROIs were especially variable. One possible explanation for variability in black ROIs is glossy feathers in several species (Maia et al. 2011), which are highly sensitive to lighting conditions, producing a white or bluish glare in intense light. Black ROIs often contain very little chromatic information, but sometimes measurement noise is amplified and interpreted as such by visual models (Schaefer et al. 2007), and this can increase variability as well. Thus, this may be a criterion to expect high variation and complex lighting effects in black ROIs when using CS photographs for studying color.

One set of research questions using color seeks to understand the role of color in the context of nonhuman visual systems. Our results suggest that CS photographs may be useful for some—but not all—of these questions. We show that CS photographs in Lab space have strong predictive power for three of the four dimensions of the two bird color spaces, and the poorly predicted dimensions include mainly information from the UV part of the spectrum. UV is not captured by the sensors in consumer cameras, and as such, only a subset of questions related to bird color vision may be addressed with CS photographs. We note, however, that variation in UV reflectance independent of variation in the rest of the visual spectrum is quite rare in birds (Andersson, 1999) and that this dimension of chromatic variation (x, *SD* = 1.42, 1.03) is much less variable than the other chromatic axes considered here (y, *SD* = 2.65, 2.37, and z, *SD* = 2.67, 2.62), which will contribute as well to weak correlations with photography data.

In our intraspecific data, the general pattern in *Macroptilium atropurpureum* shows that CS photographs have higher variability than all treatments, with the exception of “Phone flash” in RGB space only. In Lab space, this variability is mostly attributable to luminance—even with controlled lighting, standardized equipment, and standardized flash applied in this treatment. Importantly, only the achromatic component of variation exceeds that of CS photographs. Spectrometry values show the least variability in Lab space and the second least variability in RGB space. Nonetheless, we consider that overall, the results align with our hypothesis that higher control produces less imprecision.

Our research shows that in using a large sample size of uncalibrated CS photographs, comparable results can still be achieved relative to traditional spectrometry. Further, this assessment of trade‐offs and limitations will equip scientists with critical knowledge and confidence when considering the use of CS photographs in color research. Although we highlight that the use of CS photographs in studying color is highly promising, there are still some considerations in this emerging field of research. For instance, there may be saturation issues, which we found in *Senna pendula*. It will be beneficial to investigate how common this issue is in CS photographs and if there is a way to work around it. At the species level, values are strongly correlated for RGB and Lab color spaces, but we found weak correlation within the HSV color space particularly for the hue component, likely associated with hue being measured in angles and requiring circular statistics to calculate variation correctly. This suggests potential difficulties of using this color space in analyzing CS photographs. At the individual level, the differences between RGB and Lab spaces are much more evident and significant due to the separation of variation into chromatic and achromatic components, and we found that the Lab space performed slightly better in this study. We also found that a high‐quality photograph as per eBird guidelines is not a strict necessity for gleaning color data: as evidenced by the increase in standard deviation in higher quality photographs versus lower quality photographs of *Macroptilium atropurpureum*. It will likely be beneficial to have a separate set of guidelines for rating how ideal a photograph is for color analysis. The current method of extracting points of measurements in photographs manually for a large sample size can be taxing and time‐consuming. Future work in this space should look to use automated machine learning techniques such as image extraction (Ott et al. 2020) to streamline this process.

The current assumption in color research is that spectrometry produces color measurements that are the most accurate and precise. Yet, arbitrary decisions across many study setups all have an effect on the output. We have provided strong evidence that studies conducted at the lowest end of methodological control (i.e., CS photographs) can provide reliable results in the future of color research, while significantly reducing costs for data collection. With regard to analyzing color in CS photographs, we suggest, as a starting point, to measure multiple random points within the ROI of each photograph for both patterned and unpatterned patches and approach the recommended sample size of 12–14 photographs per species for interspecific studies. With future research and continuous development, it is certainly possible to further refine techniques in using CS photographs, minimize trade‐offs, and subsequently introduce it into mainstream methods of studying color.

## CONFLICT OF INTEREST

None declared.

## AUTHOR CONTRIBUTIONS

**Alexandra Laitly:** Conceptualization (equal); data curation (equal); formal analysis (equal); methodology (equal); writing–original draft (equal); writing–review and editing (equal). **Corey T. Callaghan:** Conceptualization (equal); data curation (equal); formal analysis (equal); methodology (equal); supervision (equal); writing–original draft (equal); writing–review and editing (equal). **Kaspar Delhey:** Data curation (equal); formal analysis (equal); writing–review and editing (equal). **William K. Cornwell:** Conceptualization (equal); data curation (equal); formal analysis (equal); methodology (equal); supervision (equal); writing–original draft (equal); writing–review and editing (equal).

## Supporting information

Fig S1

Fig S2

Fig S3

Fig S4

Fig S5

Fig S6

Supplementary Material

## Data Availability

The data that support the findings of this study are available in Zenodo (https://doi.org/10.5281/zenodo.4505774).
