# Supplementary figures and images for: Is color data from citizen science photographs reliable for biodiversity research?
Source: Ecol Evol. 2021 Mar 30;11(9):4071–83. doi: 10.1002/ece3.7307 (PMC8093748; doi:10.1002/ece3.7307)

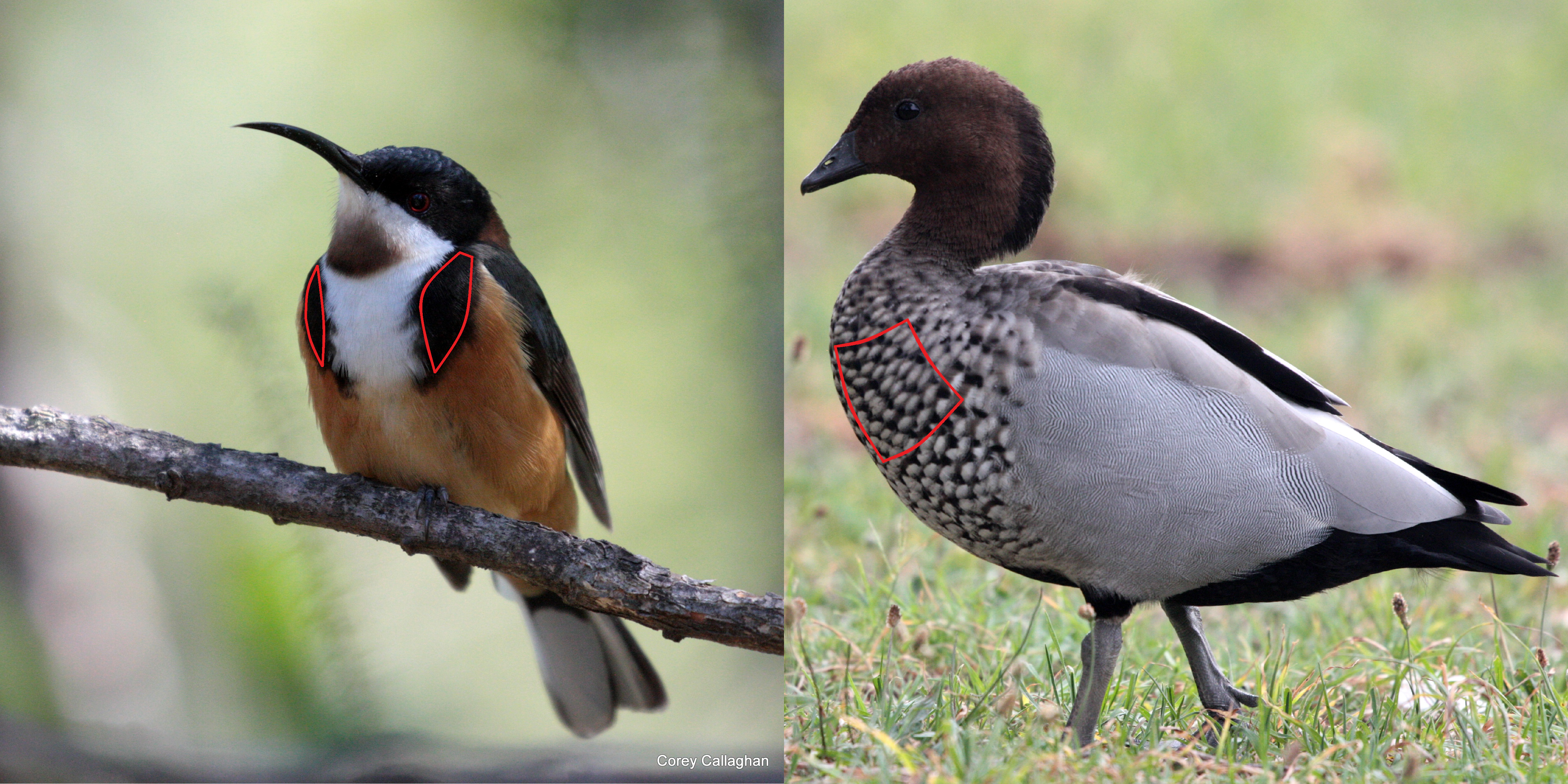

Supplement: Supplementary file 1 — Fig S1 [file ECE3-11-4071-s007.png]

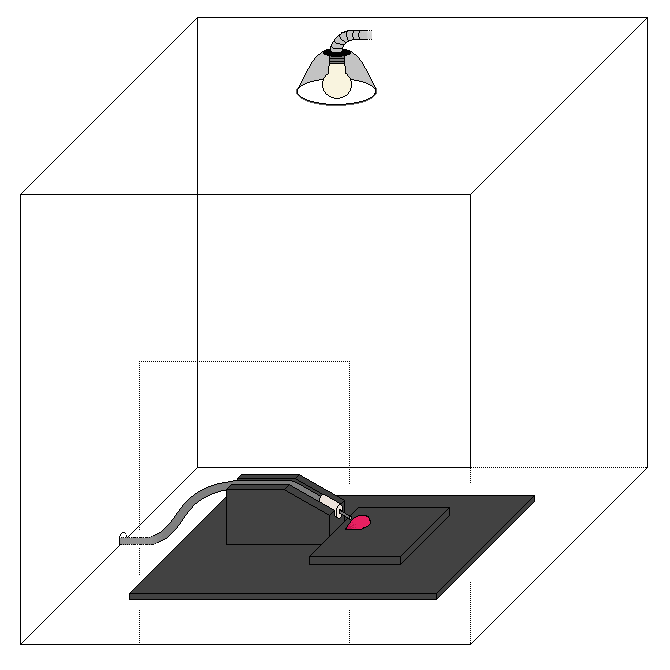

Supplement: Supplementary file 2 — Fig S2 [file ECE3-11-4071-s001.png]

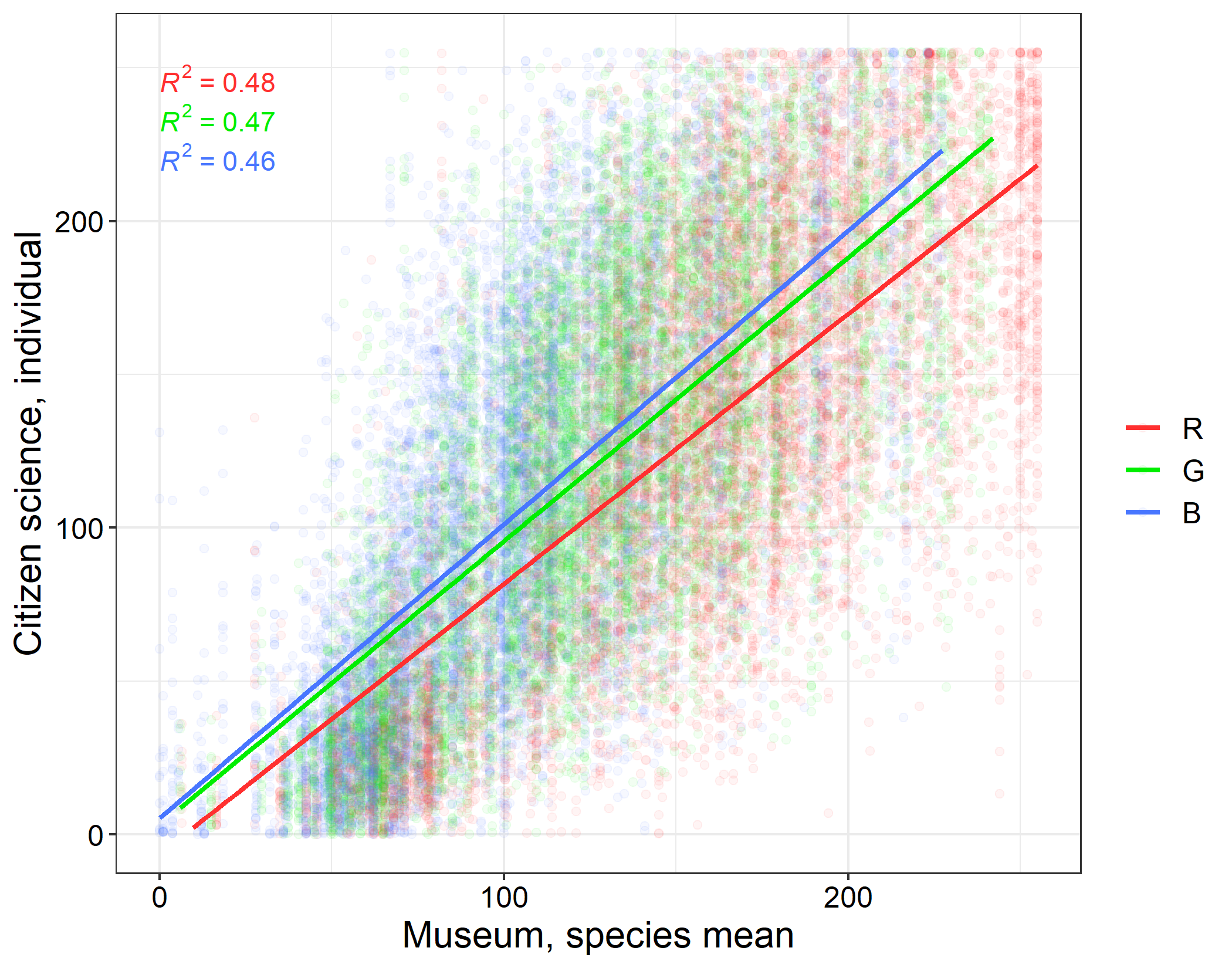

Supplement: Supplementary file 3 — Fig S3 [file ECE3-11-4071-s003.png]

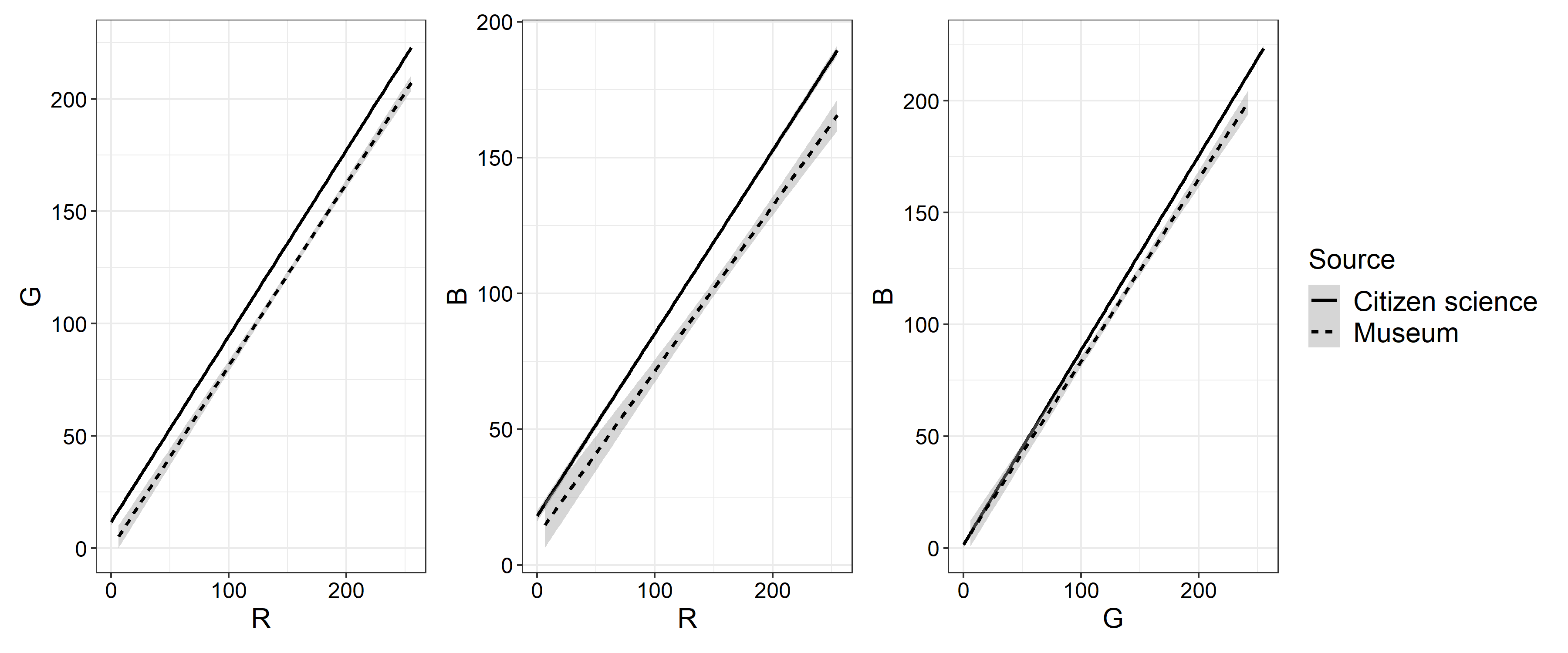

Supplement: Supplementary file 4 — Fig S4 [file ECE3-11-4071-s005.png]

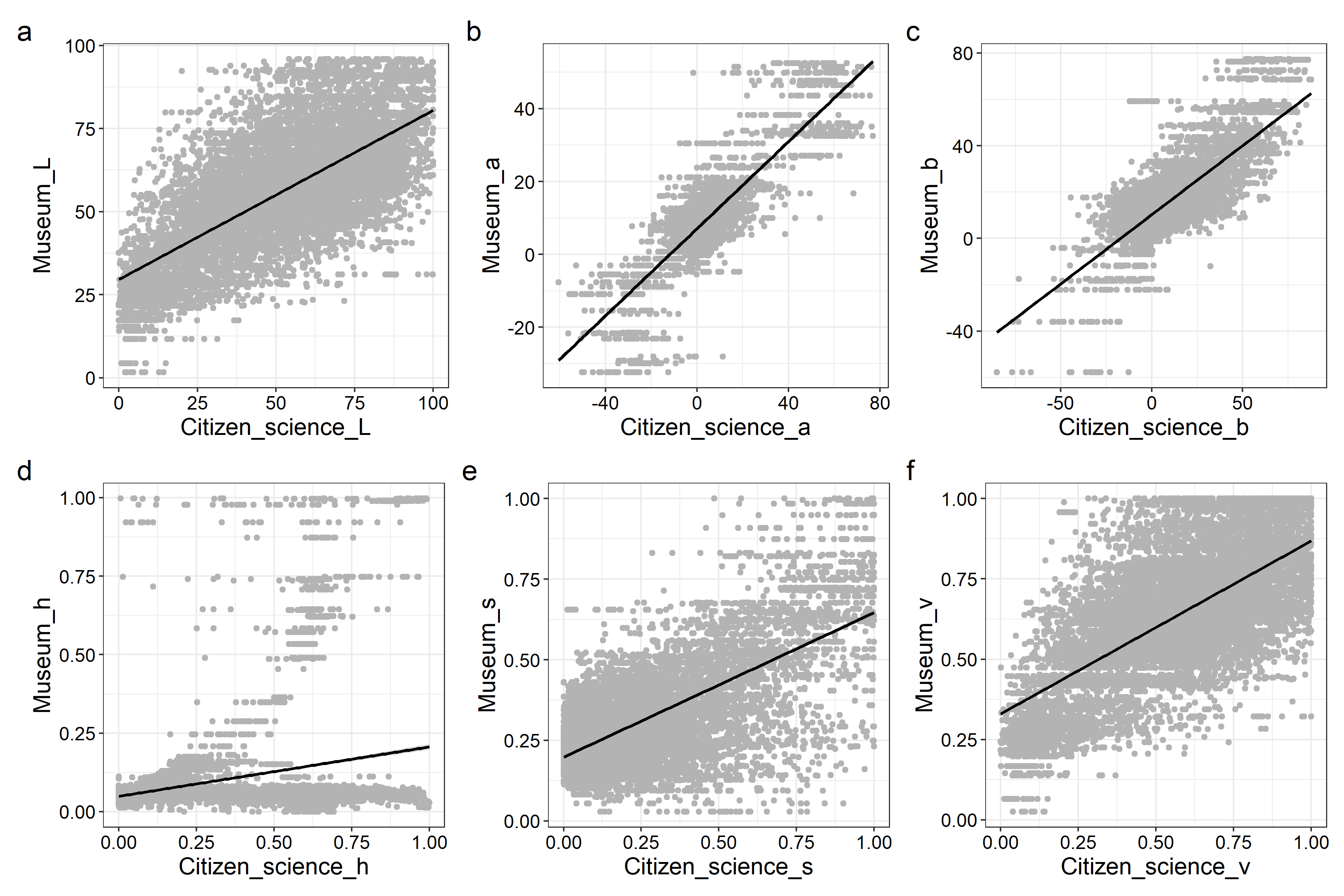

Supplement: Supplementary file 5 — Fig S5 [file ECE3-11-4071-s002.png]

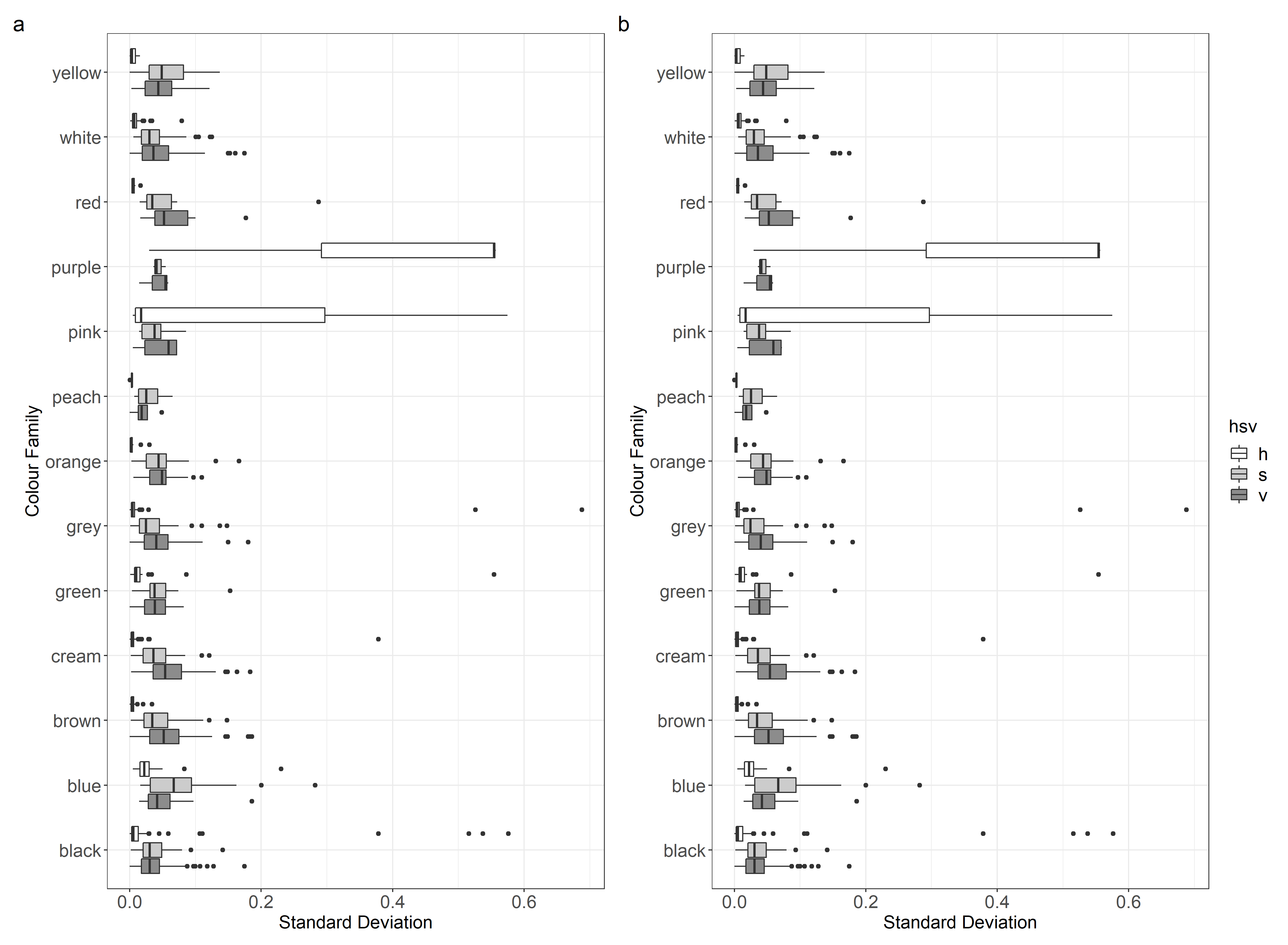

Supplement: Supplementary file 6 — Fig S6 [file ECE3-11-4071-s006.png]
