# Supplementary material for: Is color data from citizen science photographs reliable for biodiversity research?
Source: Ecol Evol. 2021 Mar 30;11(9):4071–83. doi: 10.1002/ece3.7307 (PMC8093748; doi:10.1002/ece3.7307)
Supplement: Supplementary file 7 — Supplementary Material [file ECE3-11-4071-s004.docx]

Supplementary

Supplementary Methods

A psychophysical model of avian colour vision (Vorobyev and Osorio 1998) was also used to convert reflectance spectra into chromatic and achromatic coordinates in the visual space of birds. Most birds have four types of photoreceptors, the single cones that mediate colour perception in the 300 to 700 nm wavelength range (Cuthill 2006). These single cones are sensitive to long (L, red), medium (M, green), short (S, blue) and very short (VS, near ultraviolet/violet) wavelengths of light. Achromatic cues (light-to-dark variation) is thought to be mediated by a different type of photoreceptor, the double cones (Cuthill 2006). The formulas described in Cassey et al. (2008) and Siddiqi et al. (2004) were used as implemented by Delhey et al. (2015) in the R statistical environment. Visual models require setting the following main parameters: (1) visual sensitivity functions of the photoreceptors; (2) the spectrum of the illuminant; and (3) the noise-to-signal ratios for each photoreceptor.

1. Visual sensitivity functions in birds fall into two main groups, based on their sensitivity to ultraviolet-blue wavelengths (VS and S cones): V-type and U-type eyes, the former have more limited sensitivity to UV compared to the second. Two sets of visual model results were thus computed using either V-type or U-type visual sensitivities obtained from Appendix A in Endler and Mielke (2005).
2. Given that the choice of illuminant spectrum (irradiance) usually has only limited effects on the results of visual models (Delhey and Peters 2008; Delhey et al. 2013), the spectrum of standard daylight (d65 (Vorobyev et al. 1998)) was used.
3. The noise-to-signal ratio for each single cone is computed using formula 10 in Vorobyev et al. (1998) based on the Weber fraction and the relative abundance of each type of single cone in the retina. Based on a recent review (Olsson et al. 2018), a Weber fraction of 0.1 and average relative cone proportions for all species reported in Hart (2001) were used, yielding the following noise-to-signal ratios (VS: 0.162, S: 0.12, M: 0.094, L: 0.1). For achromatic variation a Weber fraction of 0.2 (Olsson et al. 2018) was used.

Based on these visual models, cone quantum catches (i.e., how much they are stimulated by the combination of reflectance spectrum and ambient light) for the four types of single cones and for the double cone were estimated. Using formulae in Cassey et al. (2008) and Siddiqi et al. (2004), these can be transformed into one achromatic variable (DL) and a set of three chromatic coordinates (xyz) that specify the position of each reflectance spectrum in the visual space of birds which takes the shape of a tetrahedron (Vorobyev et al. 1998). In this case, variation along the z axis represents stimulation of the L cone relative to the stimulation of the VS, S and M cones; variation along the y axis stimulation of the M cone relative to S and VS; and x the stimulation of the VS cone relative to the S cone. Note that there are other projections in tetrahedral visual space where xyz coordinates align with other combinations of relative cone stimulation (eg. Endler and Mielke 2005). The xyz coordinates used here provide a suitable set of variables to compare with data from photography since two dimensions (y and z) are dominated by cones within the human visual sensitivity range (L, M, S) while x corresponds more to variation in the human-invisible UV range. We would thus expect better correlations between data derived from photographs for y and z than for x.


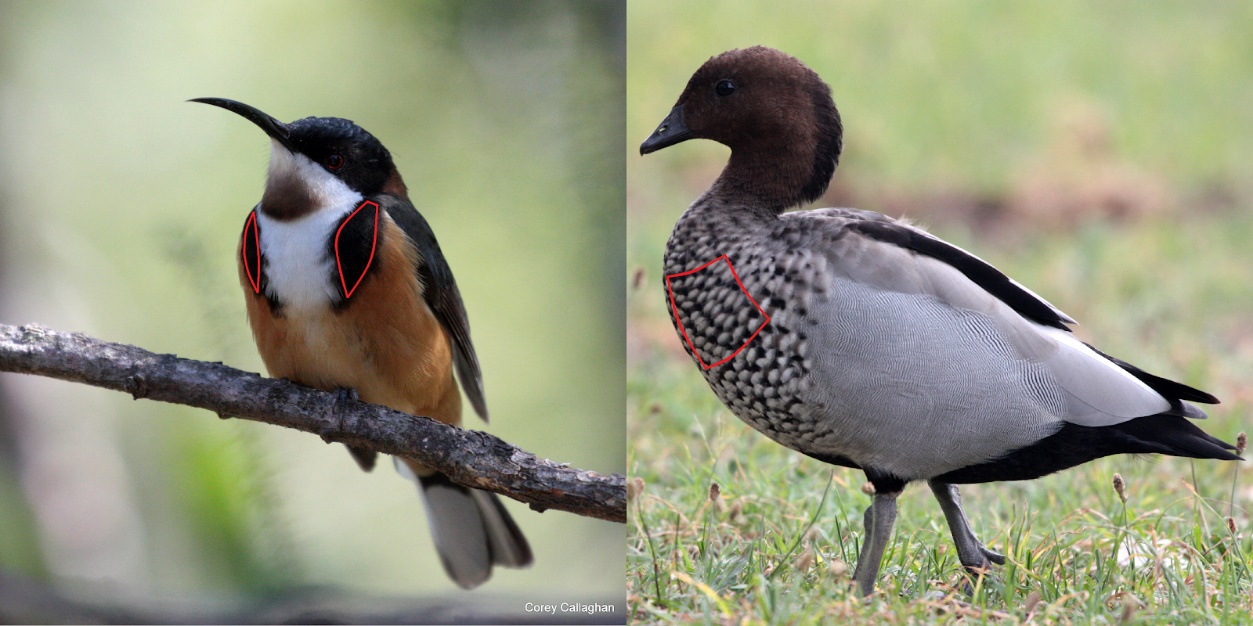


**Figure S1**. Images show different upper breast ROIs (colour-blocked, patterned) in two bird photographs, outlined in red. Photograph credit: Corey Callaghan

**Table S1**. Table showing the number of species for each colour family in descending order.

| **Colour family** | **Frequency** |
| --- | --- |
| cream | 150 |
| brown | 140 |
| grey | 117 |
| black | 102 |
| white | 94 |
| yellow | 36 |
| green | 29 |
| orange | 26 |
| blue | 16 |
| red | 10 |
| pink | 8 |
| peach | 5 |
| purple | 3 |


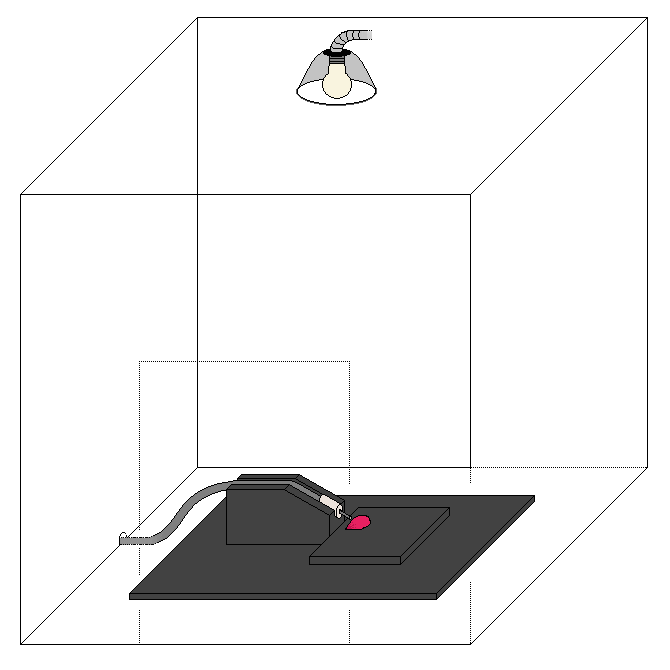


**Figure S2**. Diagram showing box setup for spectrometry, including a petal as an example.


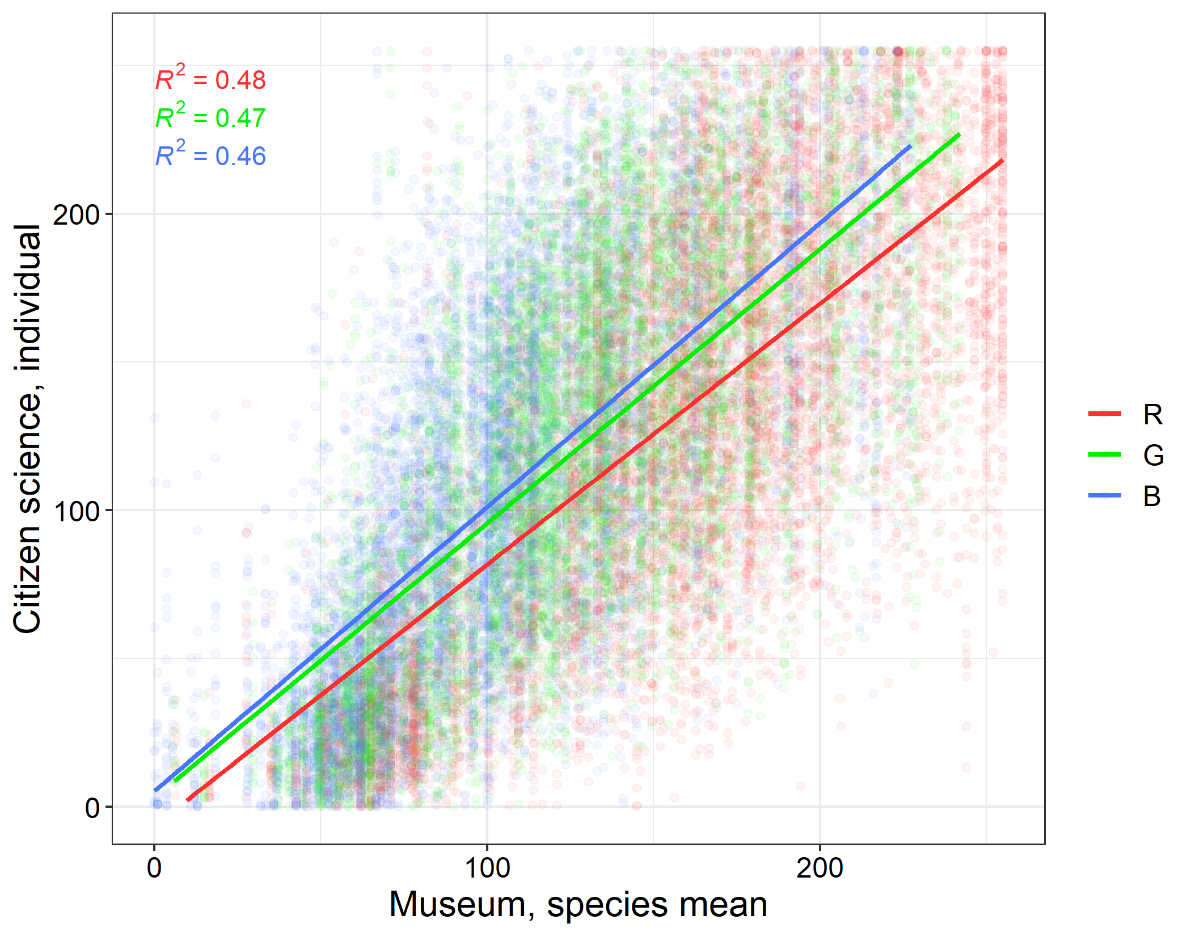


**Figure S3**. Linear model of the relationship between citizen science and museum colours in RGB space. Each point is an individual measurement in citizen science data plotted against the corresponding species mean in museum data.


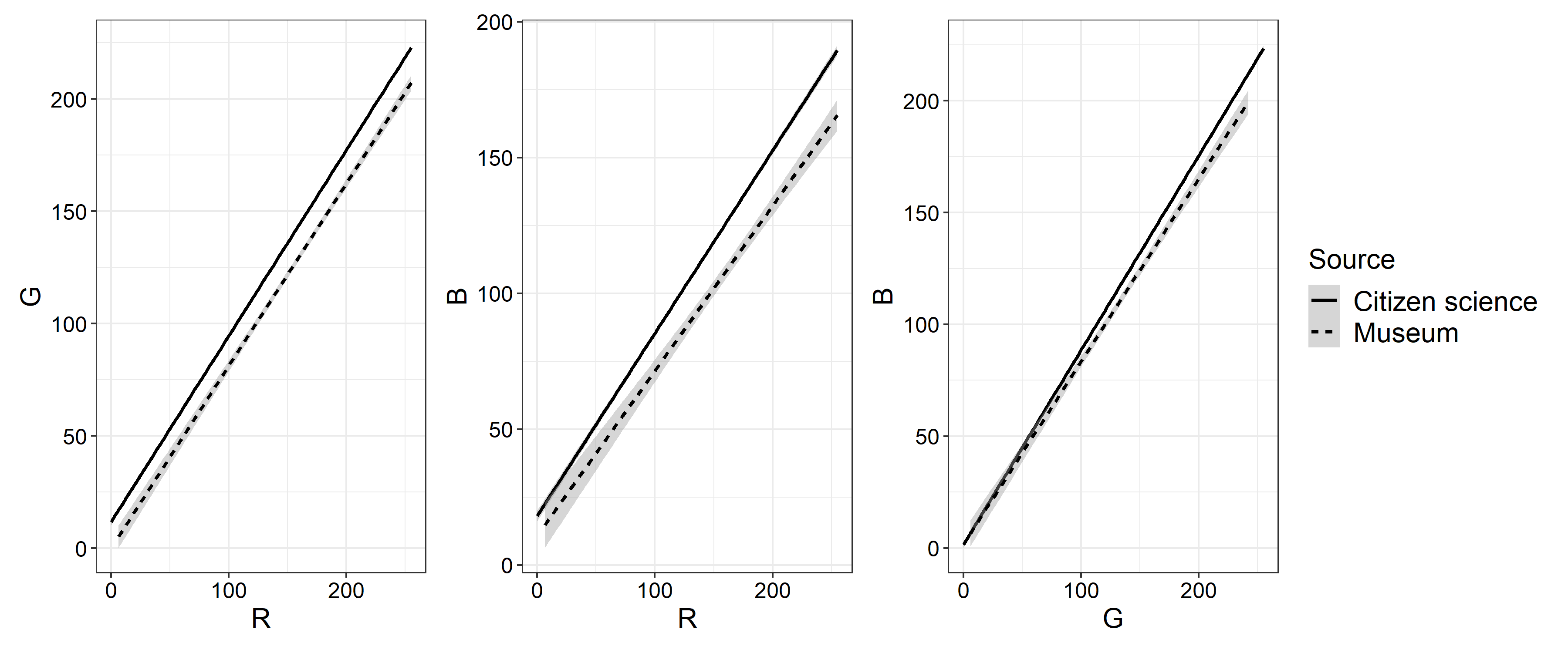


**Figure S4**. Alternative plots showing overall differences in RGB measurements between citizen science and museum data at individual level.


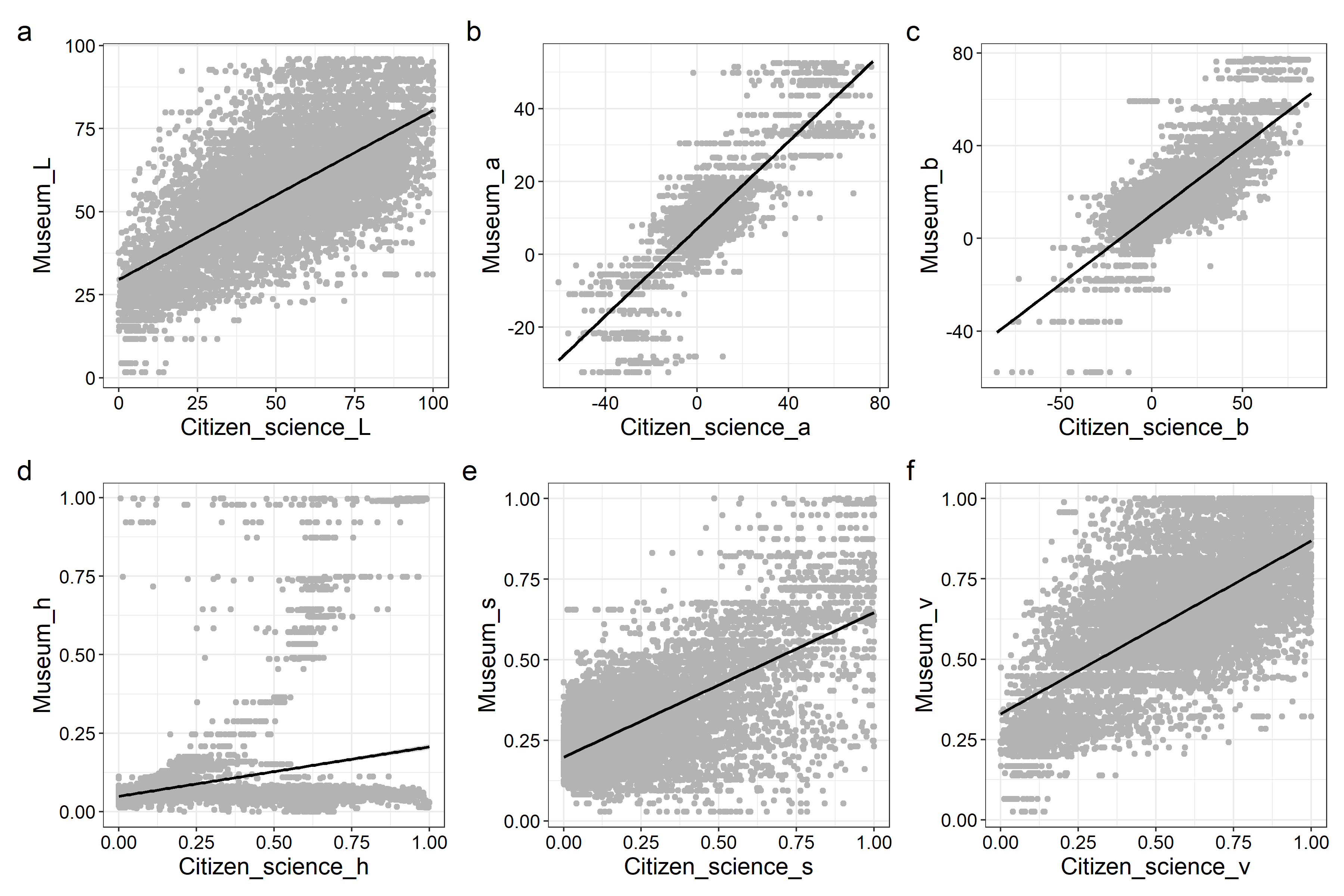


**Figure S5**. (a - c) Plots showing the relationship between museum and citizen science colours in Lab colour space at individual level. (d - f) Plots showing the relationship between museum and citizen science colours in HSV colour space at individual level.


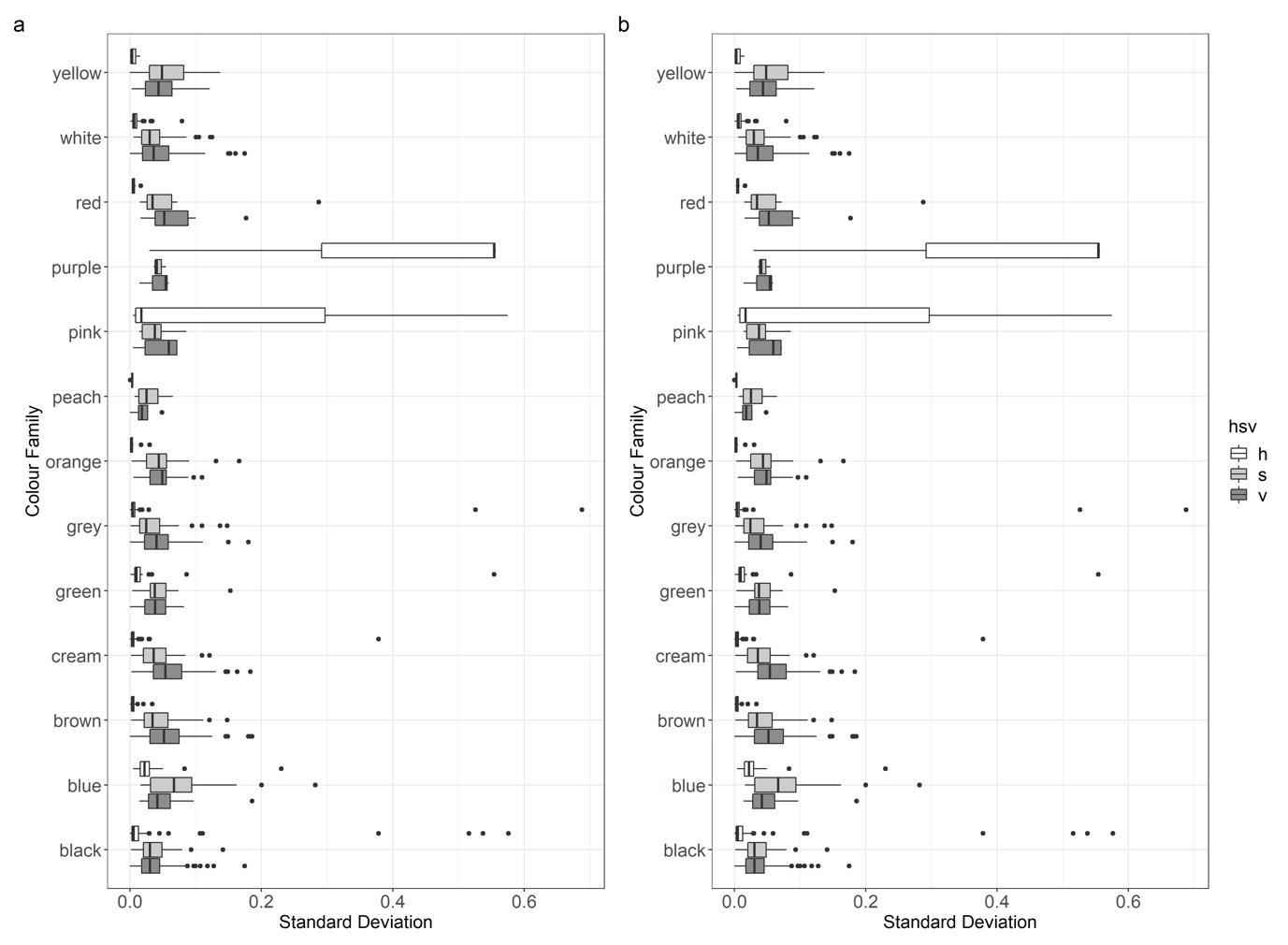


**Figure S6**. Boxplots showing differences in variability by colour family in HSV space, for (a) citizen science data, and (b) museum data. Standard deviations were calculated at species level.

### References

Cassey, P., J. Ewen, T. Blackburn, M. Hauber, M. Vorobyev, and N. Marshall. 2008. Eggshell colour does not predict measures of maternal investment in eggs of *Turdus* thrushes. *Naturwissenschaften* 95:713–721.

Cuthill, I. C. 2006. Color Perception. Pages 3-40 in G. E. Hill, K. J. McGraw, editors. Bird Coloration. Harvard University Press, Cambridge, Massachusetts.

Delhey, K., V. Delhey, B. Kempenaers, and A. Peters. 2015. A practical framework to analyze variation in animal colors using visual models. *Behavioral Ecology* 26:367–375. <https://doi.org/10.1093/beheco/aru198>

Delhey, K., M. Hall, S. A. Kingma, and A. Peters. 2013. Increased conspicuousness can explain the match between visual sensitivities and blue plumage colours in fairy-wrens. *Proceedings. Biological sciences / The Royal Society* 280:20121771. https://doi.org/10.1098/rspb.2012.1771

Delhey, K., and A. Peters. 2008. Quantifying Variability of Avian Colours: Are Signalling Traits More Variable? (Variability of Bird Colours). *PLoS ONE* 3:e1689. https://doi.org/10.1371/journal.pone.0001689

Endler, J. A., and P. W. Mielke. 2005. Comparing entire colour patterns as birds see them. *Biol. J. Linnean Soc*. 86:405-431. https://doi.org/10.1111/j.1095-8312.2005.00540.x

Hart, N. 2001. Variations in cone photoreceptor abundance and the visual ecology of birds. *Sensory, Neural and Behavioral Physiology* 187:685–697.

Johnsen, S. 2016. How to measure color using spectrometers and calibrated photographs. *Journal of Experimental Biology* 219:772–778.

Olsson, P., O. Lind, A. Kelber, and L. Simmons. 2018. Chromatic and achromatic vision: parameter choice and limitations for reliable model predictions. *Behavioral Ecology* 29:273–282. https://doi.org/10.1093/beheco/arx133

Siddiqi, A., T. W. Cronin, E. R. Loew, M. Vorobyev, and K. Summers. 2004. Interspecific and intraspecific views of color signals in the strawberry poison frog *Dendrobates pumilio*. *The Journal of experimental biology* 207:2471.

Vorobyev, M., and D. Osorio. 1998. Receptor noise as a determinant of colour thresholds*. Proceedings of the Royal Society B: Biological Sciences* 265:351–358. https://doi.org/10.1098/rspb.1998.0302

Vorobyev, M., D. Osorio, A. T. D. Bennett, N. J. Marshall, and I. C. Cuthill. 1998. Tetrachromacy, oil droplets and bird plumage colours*. Sensory, Neural and Behavioral Physiology* 183:621–633.
